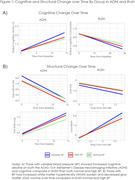# Beyond Hypertension: Examining Variable Blood Pressure’s Role in Cognitive Decline and Brain Structure Changes, Longitudinal Insights From RUSH and ADNI

**DOI:** 10.1002/alz.085922

**Published:** 2025-01-03

**Authors:** Cassandra Morrison, Michael Oliver, Farooq Kamal, Mahsa Dadar

**Affiliations:** ^1^ Carleton University, Ottawa, ON Canada; ^2^ Belmont University, Nashville, TN USA; ^3^ Douglas Mental Health University Institute, McGill University, Montreal, QC Canada; ^4^ Douglas Mental Health University Institute, Montreal, QC Canada; ^5^ McGill University, Montreal, QC Canada

## Abstract

**Background:**

High blood pressure (BP) is one of the twelve modifiable risk factors that contribute to 40% of dementia cases that could be delayed or prevented. Although high BP is associated with cognitive decline and structural brain changes, less is known about the long‐term association between variable BP and cognitive/brain changes. This study was designed to examine the relationship between variable BP and longitudinal cognitive, post‐mortem neuropathology, white matter hyperintensity (WMH), gray matter (GM) volume, and white matter (WM) volume change over time.

**Methods:**

A total of 4606 participants (3429 females, mean age = 76.8) with 32776 follow‐ups from RUSH and 2114 participants (1132 females, mean age = 73.3) with 9827 follow‐ups from ADNI were included in this study. Participants were divided into one of three groups: 1) normal BP, high BP, or variable BP. Linear mixed effects examined the relationship between the groups and cognitive and structural brain changes.

**Results:**

Older adults with variable BP exhibited the highest rate of cognitive decline followed by high BP and then normal BP. Increased GM volume loss and WMH burden was also observed in variable BP compared to high and normal BP. With respect to post‐mortem neuropathology, both variable and high BP had increased rates compared to normal BP. Results were consistent across the RUSH and ADNI participants, supporting the generalizability of the findings.

**Conclusion:**

Limited research has examined the long‐term impact of variable BP on cognition and brain structure. These findings show the importance that both high and variable BP have on cognitive decline and structural brain changes. Damages caused by variable BP may reduce resilience to future dementia related pathology and increased risk of dementia. Improved treatment and management of variable BP may help reduce cognitive decline in the older adult population.